# Are older adult research participants representative of the general population? Results from 19 clinical studies at one academic research center

**DOI:** 10.1016/j.cct.2026.108388

**Published:** 2026-06-27

**Authors:** Rebecca H. Neiberg, Xiaoyan Leng, Denise K. Houston, Kristen M. Beavers, Michael E. Miller, Kimberly Kennedy, Jason Fanning, Megan B. Irby, Dalane W. Kitzman, Stephen B. Kritchevsky, Barbara J. Nicklas

**Affiliations:** a Wake Forest University School of Medicine, United States of America; b Wake Forest University, United States of America

**Keywords:** Recruitment, Research participant, Demographics

## Abstract

**Background:**

To implement strategies for enhancing research participation across a broad range of personal characteristics, additional data are needed regarding the degree of underrepresentation of specific individuals in research.

**Methods:**

This study analyzed data from 3671 older (mean age 69 yrs) individuals enrolled in 19 studies conducted from 2002 to 2023 at Wake Forest University School of Medicine. Study inclusion was limited to those with a minimum age eligibility of 50 years. Demographic data of enrolled participants were compared to 1) individuals phone screened for participation and 2) US Census data of older individuals residing in the zip codes of the catchment area.

**Results:**

There were 11 studies with screening data available, and the overall enrollment yield was 11.1% (*n* = 2207 of *n* = 19,895 screened). Those enrolled were similar in age to those who screened out, and a greater proportion of males (14.5%) versus females (12.4%) and White (22.3%) versus Black participants (19.4%) were enrolled after screening. Compared to Census estimates of older adults, individuals that were 75+ years at enrollment were underrepresented (Census 29%, enrolled 18%; PPR = 0.6), older adults with at least some college education were overrepresented (Census 24%, enrolled 80%; PPR = 3.27) and individuals who specified Black or African American ethnicity were slightly overrepresented (Census 14%, enrolled 19%; PPR = 1.31). Older adults who were 65 years or older, female, or had White ethnicity had proportional representation at enrollment.

**Conclusions:**

The research participants included across these studies had underrepresentation of adults older than 75 years and overrepresentation of college-educated and Black or African American race.

## 1. Introduction

The ethical and unbiased conduct of clinical research is crucial for advancing medical knowledge and identifying new treatments that are effective for all individuals. One factor underlying inherent bias in research is when results cannot be generalized across a different subset of the population that differ from those enrolled in the study. Thus, it is crucial that clinical studies enroll a range of individuals who represent the entire population and, importantly, represent the health condition being studied.

As a large sponsor of clinical research, the National Institutes of Health (NIH) has sought to reduce bias and improve the external validity of clinical trials through its policies, notably by emphasizing that research participants are representative of the population of interest. For example, to address the underrepresentation of older adults in research, the NIH instituted a policy in 2019 mandating that investigators include participants of all ages or specify an acceptable reason for excluding participants based on age [1]. Data from 2021 show a shift towards enrollment of more individuals over 65 years in NIH-funded research overall, though individual studies still lacked appropriate representation of older adults [2]. In addition, a 2021 Inclusion of Older Adults in Clinical Research workshop identified major barriers and recommended strategies to address these barriers for enhancing older adult participation [3]. Undoubtedly, recognition of the gap in age representation among research participants was the first step to beginning to rectify this health disparity issue.

Another challenge to reducing bias of research findings is ensuring broad study participant representation of various demographic groups, including individuals from varying social locations. This could feature individuals from a range of racial/ethnic groups, socioeconomic and health literacy attainment levels, and rural-dwelling individuals with lower inclusion than would be expected based on population estimates. NIH has instituted policies for expanding reporting of racial/ethnic characteristics of study participants. However, socioeconomic status (SES), education level, health literacy, living environment, and other characteristics are rarely reported [4,5], even though applicability of findings in terms of effect sizes, compliance, adverse events, and/or responses to interventions are likely to vary considerably across these characteristics. As one example, both a lower SES and lower health literacy are associated with increased risk of mobility disability and cognitive decline among older adults [6–9].

One of the major goals of the National Institute on Aging's (NIA) Strategic Directions for Research for 2020–2025 is to “*Understand health disparities related to aging and develop strategies to improve the health status of older adults in diverse populations*” [10]. The importance of including broad representation of all older adults in aging research is key to meeting this goal. Hence, a related objective is to “*Develop and implement strategies to increase inclusion of underrepresented populations in aging research*” [11]. To implement appropriate strategies for enhancing research participation of excluded population groups, additional data – and innovative engagement strategies – are needed regarding the degree of underrepresentation of these individuals in the research being conducted.

Therefore, the purpose of this study was to examine the demographics of older individuals enrolled in 19 clinical trial or observational studies conducted over the span of 22 years at an academic research center (Wake Forest University School of Medicine). All the studies were supported by the NIA-sponsored Claude D. Pepper Older American's Independence Center (OAIC) grant, with data stored in our Center's Integrated Aging Studies Databank and Repository (IASDR). First, the baseline characteristics of enrolled participants were compared to individuals who were initially screened for study participation to assess whether eligibility criteria enabled participants to approximately match the age, gender, and race of those who inquired about participation after responding to recruitment advertising. The second series of analyses focused on how well enrolled participants mirror US Census estimates of older individuals residing in the zip codes of the catchment area in terms of age, gender, education, and race. Finally, as an exploratory objective, given that recruitment methods were specified on the screening forms, differences in recruitment method success overall, and by race and sex, are reported.

## 2. Methods

### 2.1. Study identification and sample sizes

We limited study inclusion to those with a minimum enrollment of 80 participants to exclude minor pilot studies that were not reflective of typical and comprehensive recruitment efforts used in larger trials. Recruitment for the included studies was conducted from 2002 to 2023 and was supported by the Wake Forest OAIC. Consequently, all studies had a minimum age eligibility of 50 years with upper age exclusions ranging from 79 to 90 years. All studies' data were deposited in the Wake Forest Pepper Center IASDR. A total of 19 studies were identified with a total of 3671 enrolled individuals across these 19 studies [12–30]. Individual study inclusion criteria and study design aspects are reported in eTables 1a and 1b in the Supplement. Most of the studies (*n* = 14) were randomized, clinical trials of a diet or exercise intervention; three studies were drug or vitamin supplement trials and two were observational cohort studies.

Although the OAIC helped recruit participants for all 19 studies, 8 of the trials conducted their own data management and analyses and therefore did not have screening data available in the IASDR [23–30]. Thus, there were only 11 studies with demographic data of all interested potential study participants who were phone screened for participation in those studies [12–22]. The total number of individuals phone screened for participation was *n* = 19,895 in these 11 studies. The number of enrolled individuals across these 11 studies was *n* = 2207 (11% of those screened), while the number of enrolled individuals in the 8 studies without screening data in the IASDR was *n* = 1464.

### 2.2. Recruitment methods

All studies were supported by OAIC staff with experience in recruitment of older adults. Pepper Center staff either conducted the recruitment and screening activities or trained individual study staff to perform these roles. In all studies, a variety of IRB-approved recruitment strategies were used to advertise the purpose of the study and the need for volunteer participants, including: 1) local media outlets (e.g., tv, radio, newspapers), 2) mass mailing of study postcards to age-eligible individuals residing near the medical center, 3) talks/seminars and placement of flyers, 4) a Pepper Center-supported database of >20,000 older adults who provided informed consent to receive study recruitment materials or the center sponsored Vital newsletter or other newsletters, 5) online social media ads, specifically Facebook, and 6) mass mailing/emailing of letters to potentially eligible older adults selected from electronic health records (EHR). Friend recommendations or referrals were also noted as recruitment sources. All study marketing materials included a voicemail-monitored phone number and email address (some studies) for interested individuals to respond to inquire about study participation. Study staff then contacted participants to conduct phone screening to determine initial eligibility for each study.

### 2.3. Demographic data collection

On initial contact the phone screen interviewer obtained and documented verbal consent to collect demographic and health information needed to determine study eligibility. Age, recruitment method, and address were collected in all 11 studies with screening data. A subset of studies also inquired about sex (*n* = 10), race/ethnicity (*n* = 8), and education level (n = 1) on the phone screener. For the 8 studies without screening data in the IASDR, demographic data used in this paper were from study data collected at baseline in those who were enrolled.

Data were also collected on individuals who were phone screened to determine the yields of several different marketing methods. Screened participants who expressed interest in the study were asked “*How did you hear about the study*?” A single response was collected for most individuals; however, some individuals reported multiple methods. Responses with multiple methods were combined into a single category named “multiple methods.” Given that most studies used a mailing list to send postcards to potential participants as their primary method of recruitment.

### 2.4. Comparison census data

For studies with screening data available where address was collected, zip codes with at least one enrolled participant were matched with the US Census 2022 American Community Survey (ACS) 5-year estimates of percent within age group (≥65, ≥75) from the total who were age 60 or greater, percent female within age group, college education attainment (≥65 years), and race (≥65 years) and presented as unweighted and weighted by number of enrolled participants within each zip code. Estimates were weighted by zip code to ensure that zip code estimates from zip codes with larger proportions of enrolled participants contributed more to the estimate than zip codes where very few participants lived. All Census ACS estimates for gender, race, and education were limited to subsets of samples who were either 65 years and older or 75 years and older. Proportions within age group (≥65, ≥75) were calculated using the denominator of total individuals aged 60 or older to align with Pepper Center goals of targeting older adults as closely as possible.

Although the ACS produces population, demographic and housing unit estimates, the decennial census is the official source of population totals for April 1st of each decennial year. In between census years, the Census Bureau's Population Estimates Program produces and disseminates the official estimates of the population for the nation, states, counties, cities, and towns and estimates of housing units for states and counties.

### 2.5. Statistical methods

Demographics of individuals enrolled in studies with and without screening data were compared descriptively using means and standard deviations. Unweighted and zip-code weighted mean prevalences were calculated for the Census estimates and compared with characteristics of enrolled participants using the Participation to Prevalence Ratio (PPR). This calculation is computed by dividing the enrolled percentage by the Census estimated prevalence, where a range of 0.8–1.2 indicates proportional representation [31]. Odds ratios of enrollment for various recruitment methods (using recruitment by postcard as the reference category) were obtained from unadjusted logistic regression analyses and used to assess differences by recruitment method overall and to test for heterogeneity in recruitment methods by race and by sex. These exploratory analysis comparisons were deemed significant at a 0.05 level.

## 3. Results

### 3.1. Demographic data of enrolled study participants overall and by availability of screening data (Table 1; eTables 2a and 2b in Supplement)

The mean age was 68.5 years with a majority being female (63.8%), White race (77.5%) and college-educated (79.7%) for the 3671 participants enrolled across the 19 studies (Table 1). The subset of 2207 enrolled participants in the 11 studies with screening data were similar to the 1464 enrolled participants in the 8 studies without screening data as there were no meaningful differences in sex, race, or age; however, individuals from studies with screening data available had a slightly higher proportion of individuals with college or post-graduate education than enrolled participants without screening data available (college or post-graduate education: 82.5% in studies with screening data vs 72.8% in studies without screening data) (Table 1). eTables 2a and 2b in the Supplement provide study-specific tabulation of demographic data.

### 3.2. Demographic data of screened individuals and comparison by enrollment status (Table 2, eTable 3)

The demographics of the *n* = 19,895 potential participants who were phone screened for the 11 studies with screening data are shown in Table 2. The mean age was 69.7 years, and they were primarily female (68%) and White race (76%). Most studies collected education data at the time of study enrollment rather than on the phone screening form, so information about education is not available in the larger group of potential participants. Supplemental eTable 3 shows these data for each of the 11 studies separately and includes the numbers of missing data for each variable. Since some studies did not collect age, sex, race, or education on the telephone screener, there were higher rates of missing data for participants who did not become enrolled in the study.

Compared to the 11% (*n* = 2207) of individuals who were ultimately enrolled following screening, the 89% (*n* = 17,688) of those who were not enrolled, either due to loss of interest or ineligibility, were of similar age (Table 2). Participants 70 years or older had an 11.6% enrollment rate while those 75 years or older had an enrollment rate of 10.2%. Although nearly twice as many females were screened than males, males had a slightly higher enrollment rate (14.5%) than females (12.4%). There were also some differences in the enrollment rates among the 10,140 people screened that had race data available. Participants identifying as White race had a 22.3% enrollment rate, followed by those identifying as Black (19.4%), while other races had the highest enrollment rate (43.1%).

### 3.3. Comparison of demographics of enrolled participants to older adults in the catchment area (Table 3)

Census data show that from the total population age group of individuals age 60 or older, the proportion of residents in the zip codes in the Wake Forest medical center catchment area who were ≥ 65 or ≥ 75 years old was 72.4% or 29.8% (Table 3). Each of the clinical studies used for these analyses had an eligibility criterion of ≥50 years and reflect proportional representation for enrollees ≥65 years old (PPR = 1.0) and slight underrepresentation for enrollees ≥75 years old (PPR = 0.6) compared to the general population of older adults(Table 3). The percentage of participants enrolled in the clinical studies who were female for was proportional for those ≥65 and ≥ 75 years (PPR = 1.1 and 1.0, respectively). Notably, the proportion of older adults with at least some college education was well overrepresented (PPR = 3.3) compared to the general population and participants with Black or African American race were also overrepresented (PPR = 1.3). On the other hand, the enrollees with White race were proportional to the general population of older adults in the catchment area (PPR = 0.9).

### 3.4. Recruitment method frequency (Table 4)

Of the 19,895 screened, 16,513 answered a question about, “How did you hear about the study?” The most frequent method of recruitment reported was postcards (44.8%, Table 4), followed by newspaper ads (15.3%) and 6–10% mentioned flyers, letters based on EHR search, or newsletter. Multiple methods were reported by 3.8%. The most common multiple method combinations included postcard plus online, friend, tv, radio or event (*n* = 219 [35% of multiple methods]) and newspaper plus online, friend, tv, radio or event (*n* = 232 [27%]). Thus, postcards were by far the most frequently used method of marketing these studies.

### 3.5. Recruitment type yields and odds of enrollment by recruitment method (Table 4 and Fig. 1 and eFigs. 1a and 1b in the Supplement)

**Fig. 1.**
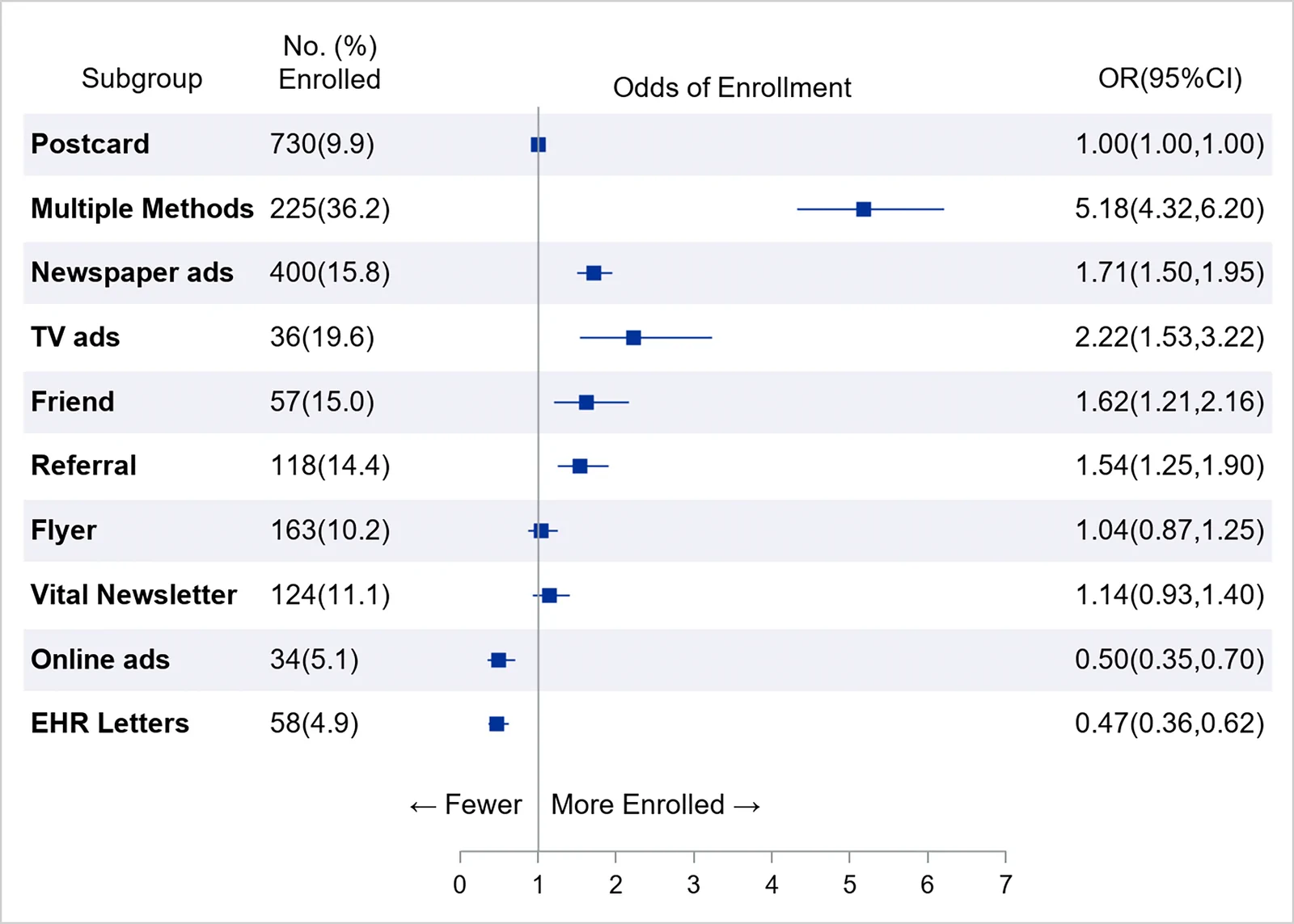
Odds of enrollment by recruitment method.

The method with the highest yield of enrollment (Table 4) was multiple (36.2%), followed by TV ads (20%), newspaper ads (16%), and friends/referrals (14–15%). Of those who reported hearing about the study through postcards, only 9.9% became enrolled. Fig. 1 shows the percentages of screened individuals who were ultimately enrolled for each recruitment method. Using postcards as the referent group because this method was the primary source used by the studies, multiple methods had five-fold greater odds of enrollment, TV ads had two-fold greater odds of enrollment, followed by newspaper, friend, and referral. Letters based on EHR search and online advertisements both had significantly lower odds of enrollment compared to post cards (Fig. 1).

In logistic regression analyses of enrollment status that investigated the heterogeneity of recruitment success by sex (interaction *P* = 0.01, eFig. 1a), the top three most successful methods for males were multiple (43%), friend (21%), newspaper ads (20%) closely followed by referral (19%). For females, multiple methods (35%), TV ads (21%), referral (21%), or newspaper ads (20%) had enrollment yields higher than postcards. When differences in recruitment method odds of enrollment were examined by race (interaction *P* = 0.049, eFig. 1b), both races had multiple methods (38% AA, 41% W) followed by referrals as the top two most successful methods (32% for both); Black/African American respondents mentioned newspaper ads (21%) or newsletter (21%) while White participants mentioned friend (27%) or television ads (26%) as third.

## 4. Discussion

There is increasing recognition of the importance of including research study participants across a broad range of personal characteristics to reduce bias and enhance generalizability of findings. This paper reports the general demographic data of 3671 older adults enrolled across 19 clinical research studies conducted at an academic medical center in Winston-Salem, North Carolina. The characteristics of the enrolled study participants were compared to 1) those who showed interest in participating and were phone screened for study participation, and 2) the demographic characteristics of the local population according to US Census estimates. The success rates of several different recruitment/marketing methods are also reported. We found that enrolled participants were similar in age with those who screened out, and that approximately 2–3% greater proportion of males (versus females) and White participants (versus Black participants) were enrolled after screening. Compared to Census estimates for adults aged 60 years or older, enrollees had proportional representation for age ≥ 65, female gender for both ≥65 and ≥ 75 year olds subsets, and White ethnicity for ≥65 year olds. However, the percentage of Black and college educated participants were overrepresented (PPR = 1.3 and 3.3, respectively) compared to the general population of adults aged 65 years or older.

We were interested in comparing the characteristics of the sample of older adults who were ultimately enrolled to the larger sample of those who underwent phone screening to determine whether specific individuals were more likely to be ineligible for study participation. The overall enrollment rate was 11.1% of those who were phone screened, which is a similar rate as other large clinical trials involving behavioral interventions in community-dwelling older adults [32–35]. Compared to individuals who were screened but not enrolled, those who were eligible and enrolled in one of the studies were about a year younger on average. Also, the enrollment rates decreased with increasing age (70+ yrs. = 11.6%; 75+ yrs. = 10.2% and 80 + yrs. = 9%), indicating that age was a demographic factor affecting eligibility despite that these were all older adults. Other studies have found that a strong barrier for under representation of older adults in clinical research is the presence of exclusion criteria that are more prevalent among older people (even in studies without an upper age limit) [3,36,37]. Thus, the age-related difference we found is likely due to a higher prevalence of specific exclusion criteria among those who were older, although further research is needed to identify these exclusionary factors.

The number of females who responded to marketing efforts and were phone screened was over twice that of the number of males who were screened. However, interestingly, the enrollment rate was higher for males than for females indicating a slightly higher screen fail rate among females. Notably, since some of the studies did not ask about race on the phone screener, the missing race data among those screened was large (49%; see eTable 3). Yet, we did find differences by race in enrollment success. Individuals with Black race had a 19.4% enrollment rate, while people with White race had a 22.3% enrollment rate. Enrollment success of individuals reporting a race other than White or Black was overly high (43.1%), likely due to the very small sample (only *n* = 72) for this group. Unfortunately, education level was only ascertained by one study at screening so the enrollment success could not be compared among those with different levels of education. Future research should attempt to ascertain and report the education, health literacy, and other socioeconomic factors of older individuals who do (or do not) respond to research marketing efforts for determining the best strategies for inclusion of all individuals in clinical research.

Our findings showed that the individuals (mean age about 69 years) who were ultimately enrolled across these 19 studies were primarily female (64%), White race (78%), and highly educated (80% with some college). Compared to the Census estimates of older adults residing in the catchment area, people who were ≥ 65 (Census 72%, enrolled 71%, PPR = 1.0) and females ≥65 or ≥ 75 were proportionally represented in the research studies (Census: 56% or 60% female, enrolled: 62% or 58% female; PPR = 1.1 or 1.0, respectively). However, for those over 75 years, the percentage of enrolled were underrepresented compared to the general population of older adults (Census: 29%, enrolled: 18%, PPR = 0.6). The percentage of those who identify as Black among those 65 and over in this area of NC is estimated to be about 14%. The proportion of our study participants with Black race was 19% and reflected an overrepresentation of this population (PPR = 1.3) that could at least partially be explained by successful efforts of WF OAIC supported studies to recruit a sample that reflected proportional representation of the racial/ethnic demographics within the catchment area. The estimates of other minority racial groups, as well as those reporting Hispanic ethnicity, among those over 65 years in the catchment area is very small (2022 Census unweighted estimate 1.7%). Previously published reports of demographic data of study participants often find that race/ethnicity are not consistently reported [38–41]. Our data showed that 49% of the individuals who were screened by phone were missing race data but that these data were collected for 100% of the individuals enrolled. On the other hand, study participants enrolled in these 19 studies were strongly disproportionately more highly educated than the general population in the area. This over representation of those with more education in clinical research has been reported by other studies and likely reflects a higher SES of individuals who volunteer and qualify for these time-intensive behavioral intervention research studies. For example, in the *n* = 1635-person LIFE study, 63.8% reported education beyond high school [32].

Given that the recruitment for all the studies included in this report was conducted in a similar manner by the Pepper Center's Recruitment Core, another goal was to determine the recruitment yields for the different recruitment/marketing methods used. Over 16,500 older adults reported how they heard about the study they were interested in during the phone screening process. Nearly half (45%) of these individuals found out about the study via a postcard mailed to their home address, which was the most frequently used recruitment strategy for most of the studies. However, the yield of eventual enrollment was not as high for the postcard method alone as other recruitment strategies, namely, using multiple methods, running TV ads, and word of mouth referrals. Interestingly, mailing letters to potentially eligible older adults selected using electronic health records (EHR) was less effective for enrollment yield than mailing postcards to age-eligible people in the area. It will be important to calculate costs of each recruitment method in the context of that method's odds of eventual enrollment success. One such study reported that direct mailings yielded more older adults for screening but was also the most expensive method per participant enrolled [42]. On the contrary, direct mailings were the most effective and least costly method for enrolling dyads in a dementia trial [43]. Like our findings, the use of the EHR to identify interested and eligible participants has been found to produce lower enrollment yields than other methods [44].

There are study design strengths of the approach we used to combine data across numerous studies to report on the representation of older adult demographic characteristics in the research conducted. One strength is the large sample size recruited from the catchment area surrounding the research center, allowing for comparisons to the Census demographic data in the same area. Another strength of the design is the ability to pool phone screening data that also included a query of the marketing strategy that yielded the greatest response from potential study participants. Finally, although not all studies collected all demographic characteristics of interest (age, race, sex, education) during the phone screening, there were very few missing data on these characteristics of those who were ultimately enrolled.

However, there are also several limitations to be kept in mind. First, since the majority of the studies included were behavioral intervention trials involving diet and/or exercise, all the study participants included in these analyses were community-dwelling and most were overweight or obese and were interested in participating in a diet or exercise study. Second, the target catchment area was Winston-Salem /Forsyth county which has its own unique characteristics which may not apply to efforts undertaken at other localities. Third, the surrounding area demographic data were obtained from the 2022 US Census ACS estimates from only one year. The recruitment periods for the studies span approximately 25 years and it is possible that the demographics of the catchment area may have changed over this time. Fourth, we are unable to determine the impact of respondents who simply chose not to participate prior to offering the demographic data needed for comparison or chose not to call the number seen on the recruitment materials. Additionally, more recent use of using the EHR to identify potentially eligible participants is a newer strategy used only in more recent studies. This may have contributed to the lower odds of enrollment for this method as potential older adult enrollees may have been hesitant to respond to EHR requests for study enrollment. Finally, there are several other participant characteristics that were not ascertained yet are important to report for ensuring broad study participant representation in future research. Among these are known social determinants of health, more specific indicators of SES (in addition to education levels), health literacy, living environment, and other characteristics such as medication use, and physical and cognitive function.

Ultimately, much greater attention needs to be given to reporting, and analyzing the impact on study results, characteristics of older adults recruited and enrolled in clinical research. Future studies should report not just age, sex, and race and ethnicity, but should attempt to identify other important characteristics of an individual that are likely to impact the external validity and generalizability of findings. Ideally, these data should be collected at the time of screening so that exclusionary factors that are preventing specific subgroups from inclusion can be identified. Data such as we report here should be expanded and analyzed in the context of how well research study participants represent the entire population and the disease or health condition being studied.

## Supplementary Material

Supplementary tables and figures

## Figures and Tables

**Table 1.** Demographics of enrolled participants overall and by screening data availability in WF OAIC supported studies 2002–2022

	Overall *19 Studies* (n=3671)	Screening Data Available *11 Studies* (n=2207)	No Screening Data Available *8 Studies* (n=1464)	

Age, mean ± SD, years	68.5 ± 6.9	68.8 ± 6.4	68.1± 7.7	
Female Sex, No. (%)				
Male	1330 (36.2%)	783 (35.5%)	547 (37.4%)	
Female	2341 (63.8%)	1424 (64.5%)	917 (62.6%)	
Race, No. (%)				
Black or African American	775 (21.1%)	461 (20.9%)	314 (21.4%)	
White	2844 (77.5%)	1715 (77.7%)	1129 (77.1%)	
Other	52 (1.4%)	31 (1.4%)	21 (1.4%)	
Education, No. (%)^a^				
No Formal Education	1 (0.0%)	0 (0.0%)	1 (0.1%)	
High school or less	547 (20.3%)	334 (17.5%)	213 (27.1%)	
College	1527 (56.7%)	1110 (58.2%)	417 (53.0%)	
Post-graduate	620 (23.0%)	464 (24.3%)	156 (19.8%)	

a Education data were missing from 4 studies (See eTables 2a and 2b)

**Table 2.** Demographics and recruitment method of screened individuals overall and by enrollment status for WF OAIC supported studies 2002–2022 with screening data

	Total Screened N	Not Enrolled N (%)	Enrolled N (%)

Overall^a^	19,895	17,688 (88.9)	2207 (11.1)
Age, mean ± SD, yrs	69.7 ± 6.9	69.9 ± 7.1	68.8 ± 6.4
Age>70 yrs (n=17,527^a^)			
<70	9892	8570 (86.6)	1322 (13.4)
70+	7635	6750 (88.4)	885 (11.6)
Age>75 yrs			
<75	14,059	12,205 (86.8)	1854 (13.2)
75+	3468	3115 (89.8)	353 (10.2)
Age>80 yrs			
<80	16,326	14,227 (87.1)	2099 (12.9)
80+	1201	1093 (91.0)	108 (9.0)
Sex, (n=16,899^a^)			
Male	5410	4627 (85.5)	783 (14.5)
Female	11,489	10,065 (87.6)	1424 (12.4)
Race, (n=10,140^a^)		7933 (78.2)	2207 (21.8)
Black or African American	2374	1913 (80.6)	461 (19.4)
White	7694	5979 (77.7)	1715 (22.3)
Other	72	41 (56.9)	31 (43.1)

*Note.* Percentages are by row for each category

a Some studies did not collect age, sex, or race on the telephone screener (see eTable 3 for missing data)

**Table 3.** Census demographic comparison with WF OAIC supported studies enrolled participants

	2022 Census ACS 5-Year Estimate^b^		Overall 19 studies n=3671 Prevalence(PPR^d^)	Studies with Screening Data 11 studies n=2207 Prevalence(PPR^d^)	Studies without Screening Data 8 studies n=1464 Prevalence(PPR^d^)
	Unweighted	Weighted^c^			

Age ≥65 years^a^	72.1%	72.4%	70.9% (1.0)	74.3% (1.0)	65.7% (0.9)
Age ≥75 years^a^	29.3%	29.8%	17.5% (0.6)	16.1% (0.6)	19.5% (0.7)
Female Sex ≥65 years	56.0%	57.1%	62.0% (1.1)	63.8% (1.1)	59.0% (1.1)
Female Sex ≥75 years	60.6%	59.9%	58.1% (1.0)	56.7% (0.9)	59.8% (1.0)
Education ≥65 years					
≥ Some College	24.4%	31.2%	79.7% (3.3)	82.5% (3.4)	72.8% (3.0)
Race ≥65 years					
Black or African American	14.17%	18.4%	18.6% (1.3)	20.3% (1.4)	15.7% (1.1)
White	82.2%	76.4%	79.9% (0.9)	78.3% (0.9)	82.6% (1.0)

a From population within catchment area age ≥ 60 years.

b Census ACS 5-year estimates include all zip codes found in the 11 studies with screening data,

c Weighted according to zip code frequency.

d PPR = Participation to Prevalence Ratio. PPRs of 0.8–1.2 reflect proportional representation. For example, enrolled percentage of 70.9% for ≥65 years is divided by census prevalence of 72.1% for a PPR of 0.98.

*Note.* Education and Race data from the Census are in those ≥ 65 years and are not provided for those ≥ 75 years

**Table 4.** Recruitment method reported by screened individuals overall and by enrollment status for WF OAIC supported studies with screening data

	Total Screened, N (%)^b^	Not Enrolled, N (% row total)	Enrolled, N (% row total)	P-value

Recruitment Method collected				<.0001
Postcard	7396 (44.8)	6666 (90.1)	730 (9.9)	
Newspaper ads	2534 (15.3)	2134 (84.2)	400 (15.8)	
Flyer	1593 (9.6)	1430 (89.8)	163 (10.2)	
WakeOne EHR letters	1189 (7.2)	1131 (95.1)	58 (4.9)	
Vital newsletter	1115 (6.7)	991 (88.9)	124 (11.1)	
Referral	818 (4.9)	700 (85.6)	118 (14.4)	
Online ads	662 (4.0)	628 (94.9)	34 (5.1)	
Multiple^a^	622 (3.8)	397 (63.8)	225 (36.2)	
Friend	379 (2.3)	322 (85.0)	57 (15.0)	
TV ads	184 (1.1)	148 (80.4)	36 (19.6)	

a More than one method reported; all other methods were reported as the only one

b Percent of those with recruitment method data (n=16,513 of 19,895 screened)

## Data Availability

Data used for these analyses are available through submitting a request on the Wake Forest OAIC IASDR website.

## Appendix A. Supplementary data

Supplementary data to this article can be found online at https://doi.org/10.1016/j.cct.2026.108388.
