# Supplementary material for: Are older adult research participants representative of the general population? Results from 19 clinical studies at one academic research center
Source: Contemp Clin Trials. Author manuscript; Available in PMC 2026 Sep 11. (PMC13563415; doi:10.1016/j.cct.2026.108388)
Supplement: Supplementary file 1 [file NIHMS2205319-supplement-Supplementary_tables_and_figures.docx]

Supplemental Materials (Online Appendix) for

**Are older adult research participants representative of the general population? Results from 19 clinical studies at one academic research center**

Rebecca H. Neiberg^1^, MS, Xiaoyan Leng^1^, PhD, Denise K. Houston^1^, PhD, Kristen M. Beavers^1^, PhD, Michael E. Miller^1^, PhD, Kim Kennedy^1^, MS, Jason Fanning^2^, PhD, Megan B. Irby^2^, PhD, Dalane W. Kitzman^1^, MD, Stephen B. Kritchevsky^1^, PhD, Barbara J. Nicklas^1^, PhD

^1^ Wake Forest University School of Medicine

^2^ Wake Forest University

Corresponding Author:

Rebecca H. Neiberg, MS

Department of Biostatistics and Data Science
Division of Public Health Sciences

Wake Forest University School of Medicine
525@Vine, 3107

Winston-Salem, NC 27157
Email: Rebecca.Neiberg@AdvocateHealth.org

| Number | Title |
| --- | --- |
| eTable 1a | Listing of included WF OAIC supported studies 2002-2022 with phone screening data available (n=11 studies) |
| eTable 1b | Listing of included WF OAIC supported studies 2002-2022 without phone screening data available (n=8 studies) |
| eTable 2a | WF OAIC supported study demographics of enrolled participants in studies with phone screening data available |
| eTable 2b | WF OAIC supported study demographics of enrolled participants in studies without phone screening data available |
| eTable 3 | WF OAIC supported study data availability for studies with phone screening data available |
| eFigure 1 | Odds of Enrollment by Recruitment Method and Sex |
| eFigure 2 | Odds of Enrollment by Recruitment Method and Race |

eTable 1a. Listing of included WF OAIC supported studies 2002-2022 with phone screening data available (n=11 studies)

| **STUDY**  **(Recruitment  dates)** | **NIH Grant #**  **Clin. trials.gov** | **Interventions (duration)** | **Eligibility Age** | **General Inclusion Criteria** | **Number Phone Screened** | **Number Rand-omized** | **Minor-ity (%)** | **Female (%)** |
| --- | --- | --- | --- | --- | --- | --- | --- | --- |
| TRAIN(12) (2002-2003) | R01 HL68901  NCT0051389 | ACE inhibitor or Placebo (6 mo.) | 55+ | High cardiovascular disease risk | 399 | 294 | 26 | 43 |
| IDEA(13)  (2006­-2010) | R01 AR052528  NCT00381290 | CR, AT/RT or CR+ AT/RT (18 mo.) | 55+ | Overweight/Obesity with knee osteoarthritis | 3035 | 454 | 18 | 72 |
| CLIP(14)  (2006-2009) | R01 HL076441  [NCT00119795](https://clinicaltrials.gov/ct2/show/NCT00119795) | Control, AT, CR+AT  (18 mo.) | 60-79 | Overweight/Obesity with cardiovascular disease | 683 | 288 | 18 | 67 |
| INFINITE(15)  (2009-2014) | R01 HL093713  [NCT01048736](https://clinicaltrials.gov/ct2/show/NCT01048736) | AT or CR+AT (5 mo.) | 65-79 | Obesity and sedentary with no aerobic exercise | 1313 | 180 | 27 | 76 |
| EVIDENCE(16) (2013-2017) | [R01 AG042411](https://www.sciencedirect.com/science/article/pii/S0002916523473509?via%3Dihub#gs1)  NCT02015611 | 2000 IU/d [Vitamin D](https://www.sciencedirect.com/topics/agricultural-and-biological-sciences/vitamin-d)_3_ or Placebo (12 mo.) | 65-90 | Low vitamin D status with low physical function | 2375 | 136 | 32 | 49 |
| MEDIFAST(17)  (2015-2016) | K01 AG047921  [NCT02730988](https://clinicaltrials.gov/ct2/show/NCT02730988) | High ProCR or Control (6 mo.) | 65-79 | Obesity with low physical function | 866 | 96 | 28 | 74 |
| EMPOWER(18)  (2016-2019) | [R01 AG051624](https://pubmed.ncbi.nlm.nih.gov/?sort=date&term=R01+AG051624%2FAG%2FNIA+NIH+HHS%2FUnited+States%5BGrants+and+Funding%5D&sort_order=desc)  [NCT02923674](https://clinicaltrials.gov/ct2/show/NCT02923674) | CR+AT or CR+SL or CR+AT+SL (18 mo) | 65-85 | Overweight/  obesity | 1656 | 183 | 26 | 77 |
| UPLIFT(19)  (2017-2020) | R01 AG050656  NCT03074643 | High ProCR+AT or RDA ProCR+AT (18 mo.) | 65-85 | Obesity with low physical function | 2760 | 187 | 26 | 66 |
| BNET(20) (2018-2021) | R01AG052419  NCT03430427 | Observational, 30 mo. | 70+ | Community-dwelling, No body weight restriction | 1657 | 192 | 10 | 56 |
| INVEST(21)  (2020-2023) | R01 AG059186  [NCT04076618](https://clinicaltrials.gov/ct2/show/NCT04076618) | CR or CR+RT or CR+weight vest (12 mo.) | 60-85 | Overweight/  obesity | 4473 | 107 | 31 | 71 |
| HALLO-P(22) (2022-2023) | U01 AG073240  NCT05424042 | TRE or In-person CR or Remote CR (9 mo.) | 60+ | Overweight/  obesity | 678 | 90 | 16 | 62 |

*Note.* CR=Caloric Restriction; AT=Aerobic Training; RT=Resistance Training; Pro=Protein intake; SL=Sitting Less; RDA=Recommended Dietary Allowance; TRE=Time-Restricted Eating.
eTable 1b. Listing of included WF OAIC supported studies 2002-2022 without phone screening data available (n=8 studies)

| **STUDY**  **(Recruitment dates)** | **NIH Grant #**  **Clin. trials.gov** | **Interventions (duration)** | **Eligibility Age** | **General Inclusion Criteria** | **Number  Randomized** | **Minority (%)** | **Female (%)** |
| --- | --- | --- | --- | --- | --- | --- | --- |
| ADAPT(23)  (1996-1998) | P60 AG10484 | Control, CR, AT+RT, CR+AT+RT (18 mo.) | 60+ | Overweight/obesity with knee osteoarthritis | 309 | 24 | 72 |
| DEMO(24)  (2002-2005) | R01 AG020583  [NCT00664729](https://clinicaltrials.gov/ct2/show/NCT00664729) | CR or CR+AT (5 mo.) | 50-70 | Postmenopausal women with abdominal obesity | 111 | 33 | 100 |
| REACTII(25)  (2002-2005) | R01HL053755  NCT00328484 | Lifestyle activity (12 mo.) or COPD rehab (3 mo.) | 55-80 | Chronic obstructive pulmonary disease (COPD) | 176 | 18 | 46 |
| PIE II(26)  (2005-2009) | M01RR007122  [NCT00123955](https://clinicaltrials.gov/ct2/show/NCT00123955) | Spironolactone or Placebo (12 mo.) | 60+ | Heart failure with preserved ejection fraction | 78 | 30 | 53 |
| OPTIMA(27)  (2006-2007) | P30 AG021332 | CR or CR+RT (4 mo.) | 65-79 | Obesity with low physical function | 88 | 10 | 45 |
| PREDICT(28)  (2007-2011) | R01HL076438  NCT00542503 | Observational, 4 years | 55-85 | Cardiovascular risk factors for congestive heart failure | 476 | 21 | 53 |
| SECRET(29)  (2009-2013) | R01 AG18915  [NCT00959660](http://clinicaltrials.gov/show/NCT00959660) | Control, CR, AT, CR+AT (5 mo.) | 60+ | Obesity with heart failure with preserved ejection fraction | 100 | 45 | 81 |
| I’M FIT(30)  (2010-2013) | R01 AG020583  [NCT01049698](http://clinicaltrials.gov/show/NCT01049698) | RT or CR+RT (5 mo.) | 65-79 | Overweight/obesity | 126 | 14 | 56 |

*Note.* CR=Caloric Restriction; AT=Aerobic Training; RT=Resistance Training

eTable 2a. WF OAIC supported study demographics of enrolled participants in studies with phone screening data available

|  | **Overall (n=2207)** | **BNET (n=192)** | **CLIP (n=288)** | **EMPOWER (n=183)** | **EVIDENCE (n=136)** | **HALLO-P (n=90)** | **IDEA (n=454)** | **INFINITE (n=180)** | **INVEST (n=107)** | **MEDIFAST (n=96)** | **TRAIN (n=294)** | **UPLIFT (n=187)** |
| --- | --- | --- | --- | --- | --- | --- | --- | --- | --- | --- | --- | --- |
| Age, mean ± SD, years | 68.8±6.4 | 76.4±4.7 | 67.1±4.8 | 70.1±4.2 | 73.9±6.3 | 67.19±4.9 | 65.6±6.2 | 69.1± 3.6 | 67.1±4.4 | 70.2±3.7 | 65.9±7.4 | 71.6±4.3 |
| Female Sex, No. (%) |  |  |  |  |  |  |  |  |  |  |  |  |
| Male | 783 (35.5) | 84 (43.8) | 95 (33.0) | 42 (23.0) | 69 (50.7) | 34 (37.8) | 129 (28.4) | 44 (24.4) | 31 (29.0) | 25 (26.0) | 167 (56.8) | 63 (33.7) |
| Female | 1424(64.5) | 108 (56.3) | 193 (67.0) | 141 (77.0) | 67 (49.3) | 56 (62.2) | 325 (71.6) | 136 (75.6) | 76 (71.0) | 71 (74.0) | 127 (43.2) | 124 (66.3) |
| Race, No. (%) |  |  |  |  |  |  |  |  |  |  |  |  |
| Black or African American | 461 (20.9) | 18 (9.4) | 48 (16.7) | 42 (23.0) | 44 (32.4) | 13 (14.4) | 79 (17.4) | 46 (25.6) | 31 (29.0) | 26 (27.1) | 69 (23.5) | 45 (24.1) |
| White | 1715 (77.7) | 173 (90.1) | 236 (81.9) | 136 (74.3) | 92 (67.6) | 76 (84.4) | 371 (81.7) | 131 (72.8) | 74 (69.2) | 69 (71.9) | 219 (74.5) | 138 (73.8) |
| Other | 31 (1.4) | 1 (0.5) | 4 (1.4) | 5 (2.7) | 0 (0.0) | 1 (1.1) | 4 (0.9) | 3 (1.7) | 2 (1.9) | 1 (1.0) | 6 (2.0) | 4 (2.1) |
| Education, No. (%) |  |  |  |  |  |  |  |  |  |  |  |  |
| High school or less | 334 (17.5) | 27 (14.3) | 53 (18.4) | 25 (13.7) | 34 (25.0) | 9 (10.0) | 72 (15.9) | 37 (20.6) | 22 (20.6) | 21 (21.9) | -------- | 34 (18.2) |
| College | 1110 (58.2) | 116 (61.4) | 177 (61.5) | 105 (57.4) | 71 (52.2) | 52 (57.8) | 277 (61.3) | 98 (54.4) | 56 (52.3) | 56 (58.3) | -------- | 102 (54.5) |
| Post-graduate | 464 (24.3) | 46 (24.3) | 58 (20.1) | 53 (29.0) | 31 (22.8) | 29 (32.2) | 103 (22.8) | 45 (25.0) | 29 (27.1) | 19 (19.8) | -------- | 51 (27.3) |

eTable 2b. WF OAIC supported study demographics of enrolled participants in studies without phone screening data available

|  | **Overall (n=1464)** | **ADAPT (n=309)** | **DEMO (n=111)** | **IM-FIT (n=126)** | **OPTIMA (n=88)** | **PREDICT (n=476)** | **PIE II (n=78)** | **REACT II (n=176)** | **SECRET (n=100)** |
| --- | --- | --- | --- | --- | --- | --- | --- | --- | --- |
| Age, mean ± SD, years | 68.1±7.7 | 68.7±6.2 | 58.9±(5.4 | 69.9±3.7 | 70.6±3.6 | 69.4±8.1 | 71.4±7.3 | 65.9±10.0) | 67.0±5.2 |
| Female Sex, No. (%) |  |  |  |  |  |  |  |  |  |
| Male | 547 (37.4) | 86 (27.8) | 0 (0.0) | 55 (43.7) | 48 (54.5) | 226 (47.5) | 18 (23.1) | 95 (54.0) | 19 (19.0) |
| Female | 917 (62.6) | 223 (72.2) | 111 (100.0) | 71 (56.3) | 40 (45.5) | 250 (52.5) | 60 (76.9) | 81 (46.0) | 81 (81.0) |
| Race, No. (%) |  |  |  |  |  |  |  |  |  |
| Black or African American | 314 (21.4) | 69 (22.3) | 37 (33.3) | 16 (12.7) | 8 (9.1) | 88 (18.5) | 22 (28.2) | 29 (16.5) | 45 (45.0) |
| White | 1129 (77.1) | 235 (76.1) | 74 (66.7) | 109 (86.5) | 79 (89.8) | 377 (79.2) | 55 (70.5) | 145 (82.4) | 55 (55.0) |
| Other | 21 (1.4) | 5 (1.6) | 0 (0.0) | 1 (0.8) | 1 (1.1) | 11 (2.3) | 1 (1.3) | 2 (1.1) | 0 (0.0) |
| Education, No. (%) |  |  |  |  |  |  |  |  |  |
| No Formal Education | 1 (0.1) | 0 (0.0) | -------- | 0 (0.0) | 0 (0.0) | -------- | -------- | 1 (0.6) | 0 (0.0) |
| High school or less | 213 (27.1) | 89 (30.0) | -------- | 30 (23.8) | 27 (30.7) | -------- | -------- | 56 (31.8) | 11 (11.0) |
| College | 417 (53.0) | 149 (50.2) | -------- | 59 (46.8) | 48 (54.5) | -------- | -------- | 90 (51.1) | 71 (71.0) |
| Post-graduate | 156 (19.8) | 59 (19.9) | -------- | 37 (29.4) | 13 (14.8) | -------- | -------- | 29 (16.5) | 18 (18.0) |

eTable 3. WF OAIC supported study data availability for studies with phone screening data available

|  | ***Overall (n=19895)*** | ***BNET (n=1657)*** | ***CLIP (n=683)*** | ***EMPOWER (n=1656)*** | ***EVIDENCE (n=2375)*** | ***HALLO-P (n=678)*** | ***IDEA (n=3035)*** | ***INFINITE (n=1313)*** | ***INVEST (n=4473)*** | ***MEDIFAST (n=866)*** | ***TRAIN (n=399)*** | ***UPLIFT (n=2760)*** |
| --- | --- | --- | --- | --- | --- | --- | --- | --- | --- | --- | --- | --- |
| Age, mean ± SD, years | 69.7±(6.9 | 75.8±5.2 | 66.8±5.1 | 70.7± 5.4 | 73.1±7.4 | 68.5±6.1 | 64.5±8.1 | 69.1±4.8 | 67.9±6.1 | 69.5±4.5 | 66.0±7.8 | 70.9±5.5 |
| Age Missing | 2412 (12.1) | 4 (0.2) | 5 (0.7) | 62 (3.7) | 69 (2.9) | 1 (0.1) | 1160 (38.2) | 33 (2.5) | 605 (13.5) | 441 (50.9) | 0 (0.0) | 32 (1.2) |
| Female Sex, No. (%) |  |  |  |  |  |  |  |  |  |  |  |  |
| Missing | 2996 (15.1) | 23 (1.4) | 2 (0.3) | 108 (6.5) | 75 (3.2) | 3 (0.4) | 830 (27.3) | 1133 (86.3) | 635 (14.2) | 92 (10.6) | 0 (0.0) | 95 (3.4) |
| Male | 5410 (27.2) | 764 (46.1) | 195 (28.6) | 425 (25.7) | 898 (37.8) | 168 (24.8) | 610 (20.1) | 44 (3.4) | 1033 (23.1) | 241 (27.8) | 223 (55.9) | 809 (29.3) |
| Female | 11489 (57.7) | 870 (52.5) | 486 (71.2) | 1123 (67.8) | 1402 (59.0) | 507 (74.8) | 1595 (52.6) | 136 (10.4) | 2805 (62.7) | 533 (61.5) | 176 (44.1) | 1856 (67.2) |
| Race, No. (%) |  |  |  |  |  |  |  |  |  |  |  |  |
| Missing | 9755 (49.0) | 498 (30.1) | 7 (1.0) | 1473 (88.9) | 318 (13.4) | 23 (3.4) | 1232 (40.6) | 1133 (86.3) | 4366 (97.6) | 457 (52.8) | 0 (0.0) | 248 (9.0) |
| Black or AA | 2374 (11.9) | 95 (5.7) | 120 (17.6) | 42 (2.5) | 658 (27.7) | 149 (22.0) | 351 (11.6) | 46 (3.5) | 31 (0.7) | 106 (12.2) | 105 (26.3) | 671 (24.3) |
| White | 7694 (38.7) | 1054 (63.6) | 552 (80.8) | 136 (8.2) | 1395 (58.7) | 498 (73.5) | 1443 (47.5) | 131 (10.0) | 74 (1.7) | 302 (34.9) | 286 (71.7) | 1823 (66.1) |
| Other | 72 (0.4) | 10 (0.6) | 4 (0.6) | 5 (0.3) | 4 (0.2) | 8 (1.2) | 9 (0.3) | 3 (0.2) | 2 (0.0) | 1 (0.1) | 8 (2.0) | 18 (0.7) |
| Address Missing, No. (%) | 2412 (12.1) | 4 (0.2) | 5 (0.7) | 62 (3.7) | 69 (2.9) | 1 (0.1) | 1160 (38.2) | 33 (2.5) | 605 (13.5) | 441 (50.9) | 0 (0.0) | 32 (1.2) |
| Recruitment Method, No. (%) |  |  |  |  |  |  |  |  |  |  |  |  |
| Multiple^1^ | 622(3.8) | 14(0.9) | 467(69.2) | 8(0.5) | 53(2.3) | 2(0.4) | 0(0.0) | 13(1.0) | 46(1.2) | 12(3.0) | 0(0.0) | 7(0.3) |
| Postcard^1^ | 7396(44.8) | 1051(65.3) | 61(9.0) | 1002(68.1) | 277(12.2) | 318(62.7) | 54(3.3) | 91(7.2) | 2415(62.5) | 297(74.1) | 185(53.9) | 1645(67.0) |
| Newspaper ads^1^ | 2534(15.3) | 3(0.2) | 4(0.6) | 28(1.9) | 537(23.7) | 5(1.0) | 1379(83.2) | 403(32.1) | 49(1.3) | 0(0.0) | 59(17.2) | 67(2.7) |
| Flyer^1^ | 1593(9.6) | 1(0.1) | 117(17.3) | 8(0.5) | 1044(46.1) | 0(0.0) | 0(0.0) | 317(25.2) | 21(0.5) | 8(2.0) | 65(19.0) | 12(0.5) |
| WakeOne EHR letters^1^ | 1189(7.2) | 281(17.5) | 0(0.0) | 76(5.2) | 0(0.0) | 0(0.0) | 0(0.0) | 0(0.0) | 503(13.0) | 0(0.0) | 0(0.0) | 329(13.4) |
| Vital newsletter^1^ | 1115(6.8) | 92(5.7) | 1(0.1) | 153(10.4) | 157(6.9) | 35(6.9) | 108(6.5) | 114(9.1) | 255(6.6) | 28(7.0) | 4(1.2) | 168(6.8) |
| Referral^1^ | 818(5.0) | 114(7.1) | 1(0.1) | 117(8.0) | 56(2.5) | 0(0.0) | 47(2.8) | 247(19.6) | 97(2.5) | 42(10.5) | 10(2.9) | 87(3.5) |
| Online ads^1^ | 662(4.0) | 30(1.9) | 0(0.0) | 41(2.8) | 26(1.1) | 56(11.0) | 0(0.0) | 4(0.3) | 427(11.0) | 1(0.2) | 0(0.0) | 77(3.1) |
| Friend^1^ | 379(2.3) | 24(1.5) | 0(0.0) | 38(2.6) | 26(1.1) | 91(17.9) | 0(0.0) | 68(5.4) | 54(1.4) | 13(3.2) | 0(0.0) | 65(2.6) |
| TV ads^1^ | 184(1.1) | 0(0.0) | 20(3.0) | 0(0.0) | 76(3.4) | 0(0.0) | 70(4.2) | 0(0.0) | 0(0.0) | 0(0.0) | 18(5.2) | 0(0.0) |
| Event^1^ | 18(0.1) | 0(0.0) | 4(0.6) | 0(0.0) | 12(0.5) | 0(0.0) | 0(0.0) | 0(0.0) | 0(0.0) | 0(0.0) | 2(0.6) | 0(0.0) |

^1^percent of those with recruitment method reported (n=16,513 overall); Recruitment methods other than “Multiple” were the only method reported

eFigure 1a. Odds of Enrollment by Recruitment Method and Sex

eFigure 1b. Odds of Enrollment by Recruitment Method and Race
